# The B-Score is a novel metric for measuring the true performance of blood pressure estimation models

**DOI:** 10.1038/s41598-022-16527-2

**Published:** 2022-07-16

**Authors:** Tomas L. Bothe, Andreas Patzak, Niklas Pilz

**Affiliations:** Charité – Universitätsmedizin Berlin, Corporate Member of Freie Universität Berlin and Humboldt-Universität zu Berlin, Institute of Translational Physiology, Chariteplatz 1, 10117 Berlin, Germany

**Keywords:** Biological models, Biophysical methods, Software, Biophysics, Biotechnology, Computational biology and bioinformatics, Physiology, Cardiology, Diseases, Medical research, Risk factors, Signs and symptoms

## Abstract

We aimed to develop and test a novel metric for the relative performance of blood pressure estimation systems (B-Score). The B-Score sets absolute blood pressure estimation model performance in contrast to the dataset the model is tested upon. We calculate the B-Score based on inter- and intrapersonal variabilities within the dataset. To test the B-Score for reliable results and desired properties, we designed generic datasets with differing inter- and intrapersonal blood pressure variability. We then tested the B-Score’s real-world functionality with a small, published dataset and the largest available blood pressure dataset (MIMIC IV). The B-Score demonstrated reliable and desired properties. The real-world test provided allowed the direct comparison of different datasets and revealed insights hidden from absolute performance measures. The B-Score is a functional, novel, and easy to interpret measure of relative blood pressure estimation system performance. It is easily calculated for any dataset and enables the direct comparison of various systems tested on different datasets. We created a metric for direct blood pressure estimation system performance. The B-Score allows researchers to detect promising trends quickly and reliably in the scientific literature. It further allows researchers and engineers to quickly assess and compare performances of various systems and algorithms, even when tested on different datasets.

## Introduction

High arterial blood pressure (BP) levels lead to higher numbers of cardiovascular events and all-cause mortality^1,2^. Cuff-based BP measurement devices have dominated the field of arterial BP determination for over one century. There has been substantial recent interest in alternative, cuff-less and continuous BP measurement devices^1–8^. Cuff-less BP measurement has the advantage of being possibly less disturbing (night BP) and providing beat-to-beat BP data. More data points benefit secondary parameter calculation, such as the BP variability^9–14^. The growing interest manifests itself in a multitude of research papers, proposing various options for cuff-less BP estimation^15–20^.

Model performance has been assessed in a multitude of ways. Unfortunately, the performance of proposed BP estimation models is only evaluated by absolute metrics. Used absolute metrics include the mean value deviation, standard deviation, mean absolute error, root mean squared error (RMSE), and many more. Regrettably, these absolute values depend not only on the BP model’s sophistication but also on what dataset it is used on. Datasets can be very different: Compare 24 h ambulatory BP measurements of clinical patients with measurements taken at rest in a laboratory setting amongst the young and healthy. Clinical patients are a heterogeneous group and therefore BP values differ more between individual patients than between young and healthy subjects (interpersonal variability). Further, 24 h measurements are less stable than measurements taken at rest within single patients (intrapersonal variability). This results in a critical problem: A given absolute value (e.g., mean value deviation) does not provide information about the true model performance. In reverse, model performances cannot be compared when not tested on the same dataset. Absolute metrics will show better results for “easier” (lower variability) datasets. Two options remain: Testing every model on the same dataset or creating a metric able to depict dataset-adjusted performance. Option one seems to be nearly impossible due to various reasons (e.g., proposed models use very different input data)^7,21–23^. In consequence, a new metric of dataset-adjusted (relative) model performance is needed. Such metric would allow easy and reliable comparison between models even when tested on different datasets.

After realizing this lack of comparability between different BP estimation model performances, we decided to develop a metric of relative model performance, the Base-Score (B-Score). The B-Score is designed to be intuitively understandable. It allows direct and easy evaluation of model performance, as higher B-Scores equal better dataset-adjusted (relative) model performance.

We tested the B-Score on generic datasets and further provide a real-world application example of comparing two very different datasets in this work. We used a published model tested on a small dataset and set it in contrast to the MIMIC IV clinical database, the to our knowledge largest dataset available^24,25^.

## Methods

The B-Score is calculated by comparing a proposed model’s absolute performance to dataset specific base-performances (B1, B2). These base performances are: The B1-performance, measuring the interpersonal variability, the B2-performance, measuring the intrapersonal variability, and the M-performance, measuring the performance of a minimalistic BP estimation model (M). Setting the absolute performance of the researcher’s model (T) into contrast with the three base performances (B1, B2 and M) results in a novel measure of relative performance. The B-Score for systolic and diastolic estimations are calculated separately, allowing a differentiated analysis of systolic and diastolic performance.

### B-Score calculation

#### Root mean squared error (RMSE)

The B-Score to evaluates relative model performance based on absolute performance measures. The metric of absolute model performance on which the B-Score is based on is the root mean squared error (RMSE). We chose the RMSE for a specific reason: It is a measure of average differences between a researcher’s values and a reference method but also takes the reliability of those differences into account. This becomes evident when comparing the RMSE to another measure of absolute performance (e.g., the mean absolute error). Being off 4 mmHg in every measurement leads to a mean absolute error of 4.0. If every second measurement is perfectly accurate but every other measurement is off by 8 mmHg the mean absolute error stays 4.0. In scenario one the RMSE equates to 4.0 as well but changes to 5.66 in the second case. The RMSE provides additional information about the measurement consistency which is important for any BP estimation. Further, it also provides information about the absolute error, combining the advantages of pure absolute measures (e.g., mean absolute error) and pure measures of proportionality (e.g., correlation coefficient)

$$RMSE= \sqrt{\frac{1}{n} \sum_{i=1}^{n}{\left(prediction-reference \,value\right)}^{2}},$$

*n* = number of samples.

#### Test-RMSE

The Test-RMSE (T-RMSE) is the measure of absolute performance which we designed the B-Score to base upon. It is the RMSE between the BP estimations a researcher’s model derived values and the reference values provided in the study.

$$T RMSE= \sqrt{\frac{1}{n} \sum_{i=1}^{n}{\left(researche{r}{\text{'}}model \,prediction-reference \,value\right)}^{2}},$$

*n* = number of samples.

#### B1-RSME

The B1-RMSE is the measure of *inter*personal variability in the researcher’s dataset. The B1-RMSE is calculated as the RMSE between the mean of all reference BP values and every single reference value. This is closely connected to the dataset’s standard deviation but not entirely equal. The reason for choosing the RMSE are explained above.

$$B1 RMSE= \sqrt{\frac{1}{n} \sum_{i=1}^{n}{\left(cohort \,mean-reference \,value\right)}^{2}},$$

*n* = number of samples.

#### B2-RMSE

The B2-RMSE is the measure of *intra*personal variability in the researcher dataset. The first measurement for every subject is defined as their personal “calibration value”. The B2-RMSE is calculated as the RMSE between the calibration value (subject specific) and every single reference value. This equates to estimating a subjects first measurement result for every upcoming measurement.

$$B2 RMSE= \sqrt{\frac{1}{n} \sum_{i=1}^{n}{\left(\text{``}calibration\text{''}\, value-reference \,value\right)}^{2}},$$

*n* = number of samples.

#### M-RMSE

The M-RMSE is the measure of how easy it is to derive a well-performing BP estimation model for the researcher’s dataset. To assess this, we created a minimalistic Deep Learning architecture. This architecture does not change but must be retrained for every given dataset (as well as for systolic and diastolic values). We designed the architecture to ensure reliable results and therefore applicability for almost all datasets.

**Figure 1:**
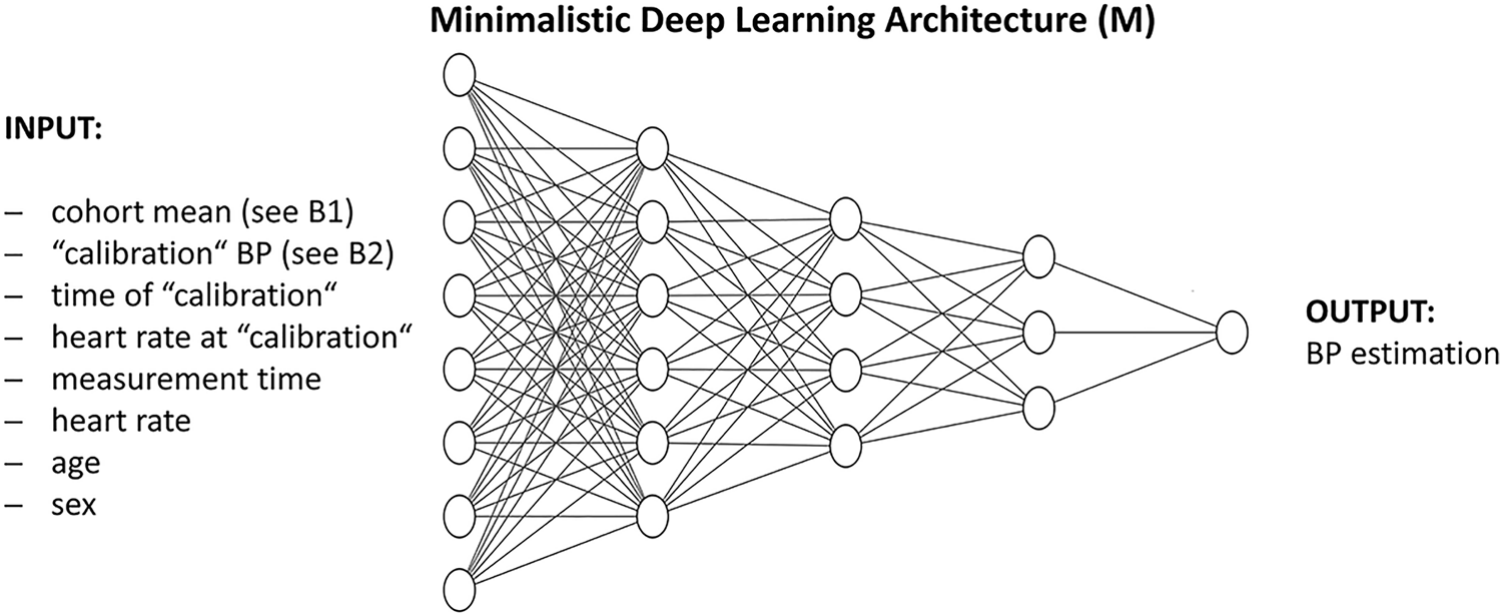
Minimalistic Deep Learning architecture used for M-RMSE calculation. It is a five-layer feed-forward Neural Network with Dropout and L2-Regression for ensuring reliable results. The system must be retrained for every dataset and provides BP estimations which are needed to M-RMSE calculation. For further information please see Supplementary Appendix 1/2.

This minimalistic tool intakes parameters present in every BP dataset: The subjects age, sex, the time of measurement, the heart rate, a calibration BP (see B2-RMSE), the time of calibration, the calibration heart rate, and the mean of all reference values (see B1-RMSE). The system then estimates BP values (Fig. 1).

The M-RMSE is calculated as the RMSE between the system’s estimations and every single reference value. Detailed insight into the Deep Learning architecture can be found in the Supplementary Appendix and the provided code (Supplementary Appendix 1/2).

$$M RMSE= \sqrt{\frac{1}{n} \sum_{i=1}^{n}{\left(minimalistic\, model\, predictions-reference\, value\right)}^{2}},$$

*n* = number of samples.

As the M-RMSE is only based on a single BP calibration and subsequent monitoring of heart rate and time, it would be easily implemented using a singular cuff measurement and subsequent “smartwatch” monitoring. The M-RMSE is therefore a reasonable minimal standard for any BP estimation model to beat.

#### B-Score

To retrieve a measure of relative performance the B-Score sets the T-RMSE (absolute performance of researcher’s model) in relation to the three presented, dataset-specific RMSE values (B1, B2, M). The B-Score is designed to increase with increasing model performance. To achieve this, it rises the more the tested model (T-RMSE) outperforms the base performances (B1-, B2- and M-RMSE):

$$B Score= {\text{log}}_{10}\left(\sqrt{\left(\frac{B1 RMSE \cdot M\, RMSE}{{T \,RMSE}^{2}}\right) \cdot \left(\frac{B2 \,RMSE \cdot M \,RMSE}{{T\, RMSE}^{2}}\right)}\right).$$

We defined the B-Score for all instances in which the T-RMSE is smaller than the M-RMSE. If this is not the case the proposed model performs worse than the minimalistic Deep Learning architecture and possibly worse than B1 or B2. We recommend to report “B-Score < 0.00” for every case that the T-RMSE is greater than any base performance (B1-, B2-, M-RMSE). The B-Score should be reported rounded to two decimal places.

A minimum of three patients with at least three measurements per patient are needed for B-Score calculation. We do not recommend calculating the B-Score for datasets containing less than 100 distinct BP measurements to ensure reliable results.

#### Reliable B-Score results

In line with the best practices of Machine Learning the M-RMSE is calculated via k-fold evaluation. To ensure reliable results, the calculation is repeated, resampled, and averaged, depending on the datasets size. In some cases, the M-RMSE might be larger than other base performances because of measures taken to ensure generalized model behaviour (e.g., Dropout, L2-regularization). Specific information is available in the provided code (Supplementary Appendix 2).

### Testing the B-Score for desired properties

We tested the B-Score to confirm reliable results and desired properties of identifying superior BP estimation systems on generic datasets.

### Dataset generation

We created three datasets (3 × systolic + diastolic) to test the B-Score. The datasets display basic characteristics of BP profiles. Short-time and long-time fluctuation rhythms primarily influence BP fluctuations, which themselves are based on the Traube-Hering-Mayer- and circadian rhythms^26–28^. Based on these rhythms we modelled two (2 × systolic + diastolic) 24-h BP datasets. The datasets differ in inter- and intrapersonal variability and are therefore named “Normal 24-h” and “Hard 24-h” datasets. We further modelled one dataset (systolic + diastolic) to be a 30-min dataset, replicating a laboratory measurement setting. It is named the “Lab” dataset. Additional information about the dataset generation is presented in the Supplementary Appendix 3.

**Figure 2:**
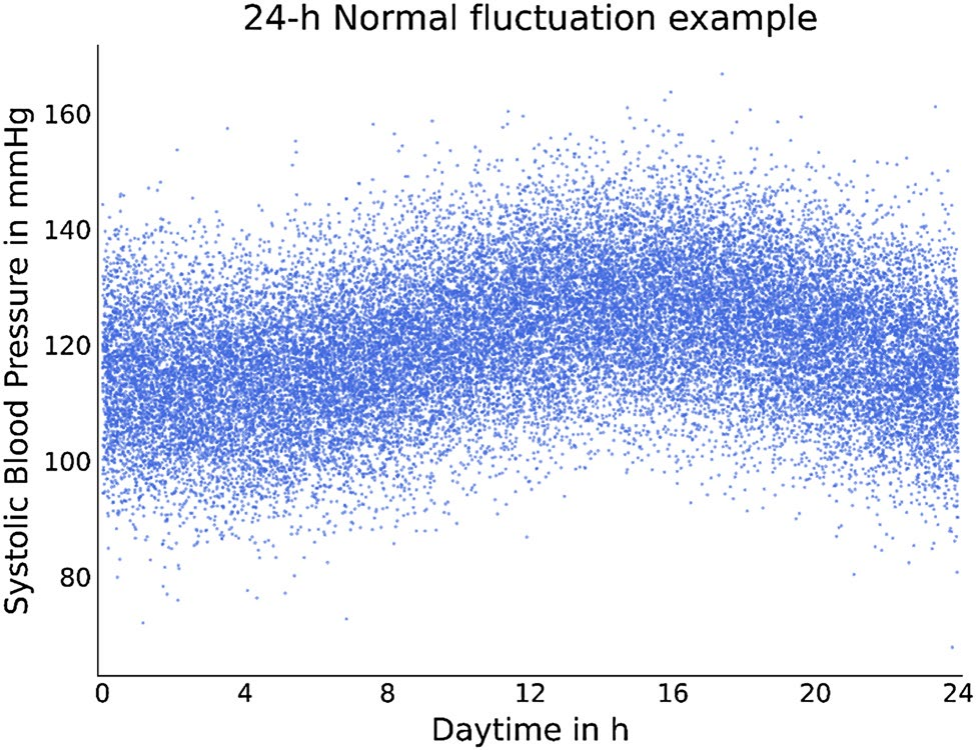
Plot of 30,000 samples from the systolic “24-h Normal” dataset. The normally distributed circadian rhythm is displayed.

For every dataset, 10,000 subjects with 50 measurements each were simulated, resulting in datasets with 500,000 single measurement entries (Fig. 2).

We set a fictional T-RMSE value of 4 mmHg and calculated the B-Score accordingly for all six datasets. The B-Score can be considered functional if calculation is trouble-free and the retrieved B-Scores clearly rank the datasets from lowest (easiest, “Lab") to highest (hardest, “24-h Hard”).

#### B-Score under increasing standard deviation

We further simulated smaller (50,000 BP values) generic datasets with increasing BP standard deviation. Followingly, we calculated the base performances (B1-, B2- and M-RMSE) and B-Scores for all datasets and plotted the results against the BP standard deviation.

### Published small dataset

We used a small dataset published by Patzak et al. in 2015 to test the B-Score at the lower boundary of dataset size. The dataset consists of 12 patients with a total of 107 (each systolic and diastolic) measurements. The authors validated a pulse-wave-velocity-based BP estimation device against intraarterial measurements taken during dobutamine induced BP increases^20^. We calculated the systolic and diastolic B-Score for the proposed device and dataset.

### MIMIC IV dataset

The MIMIC IV clinical dataset is the latest iteration of the to our knowledge largest available clinical dataset providing BP data. Data are descended from mainly ICU patients^24,25^. We used the MIMIC IV dataset to stress test the B-Score for applicability for the largest available dataset.

#### Data cleaning

We pre-processed the dataset to only hold data point suitable for the B-Score. Specifically, we kept data points which provided BP information as well as information about the additional input parameters (time of measurement, heart rate, sex, age) the M-RMSE requires. We kept BP data points within 3 standard deviations of the mean (= 99.8%) to mitigate the effect of stray-bullet measurements. We reduced the MIMIC IV dataset from nearly 330 million data points to a systolic and diastolic dataset (> 2.3 million entries each).

#### B-Score interpretation

We calculated the B1-, B2- and M-RMSE for both the systolic and diastolic MIMIC IV dataset. We were then able to interpret the results from the dobutamine-dataset in respect to the MIMIC IV dataset. More specifically, we were able to calculate a T-RMSE value of equal B-Scores. Reaching this T-RMSE value (on the MIMIC IV dataset) coequals the system performance proposed by Patzak et al.^20^. The calculation is easily obtained by transposing the B-Score equation. It is available in the Supplementary Appendix and directly computed in the provided code (Supplementary Appendix 2/4).

#### Time complexity analysis

We split the MIMIC IV dataset into smaller subsets to analyse the time needed for B-Score calculations depending on the dataset size. The calculation was performed on a single core of an Intel i9 12900K CPU.

### Programming packages and code

#### Programming packages

We wrote our programs in Python 3 (3.7.10) and primarily used the NumPy (1.19.5) and pandas (1.1.5) libraries for dataset cleaning and processing^29,30^. For model creation and evaluation, we relied on the Tensorflow2 (2.4.1) and sklearn (0.22.2) libraries^31,32^. We used the Matplotlib (3.2.2) library and NN-SVG for visualization^33,34^.

#### Code

The code for B-Score calculation is provided as an Supplementary Appendix to this article (Supplementary Appendix 2).

## Results

### B-Score test with generic datasets

The created development datasets showed expected normal distributions, with highest variability in the “24-h Hard” and lowest in the “Lab” dataset (Supplementary Appendix 5).

**Figure 3:**
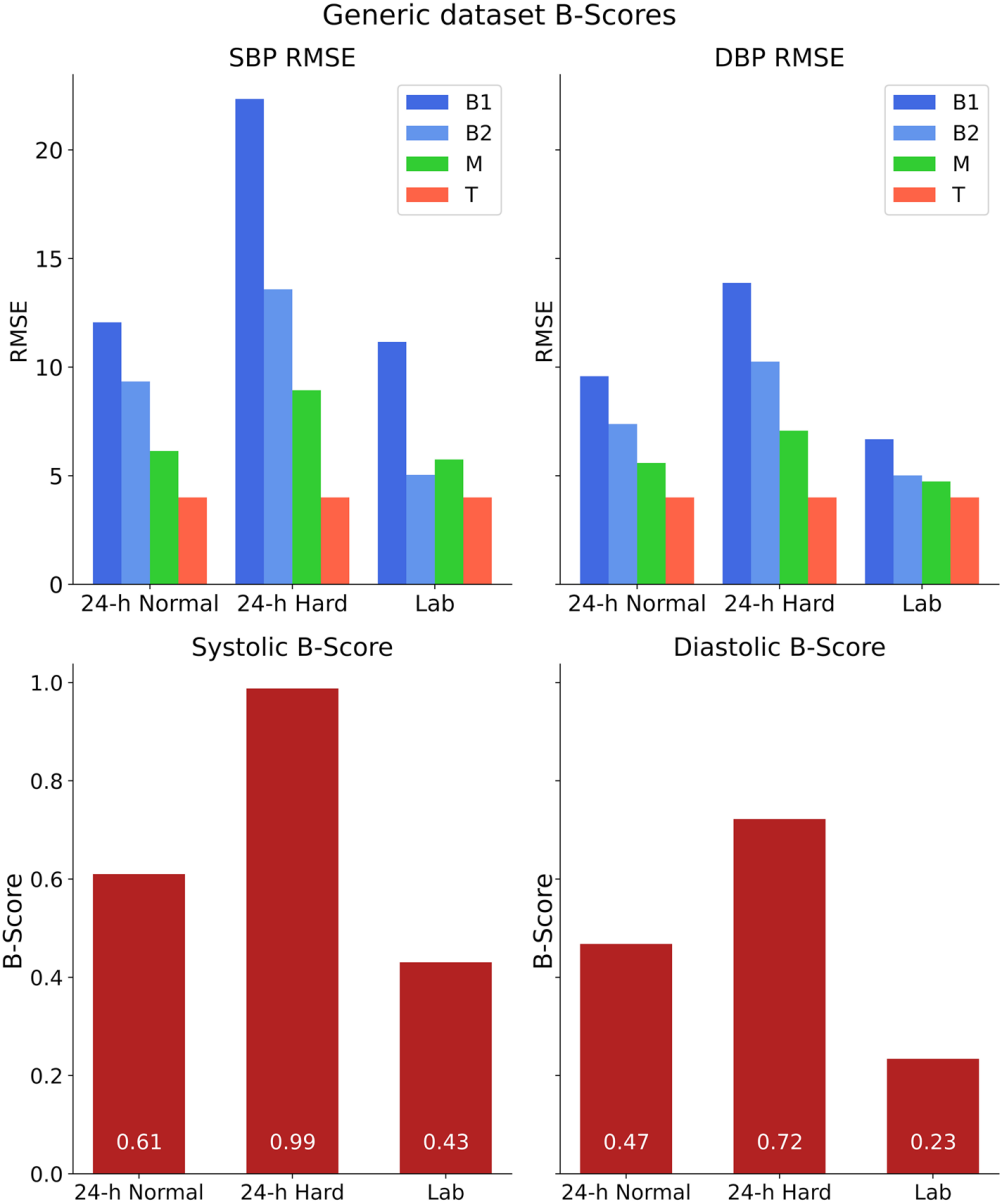
RMSE values (upper panel) and calculated B-Scores (lower panel) for all systolic (left) and diastolic (right) generated datasets. As expected, the “24-h Hard” dataset generated the highest B-Score for a fictional T-RMSE. The RMSE values indicate differences in dataset complexity (“Lab” easy to “24-h Hard” hard) which are reflected in the associated B-Scores.

We calculated the base performances (B1-, B2-, M-RMSE) values for each of the six datasets. We then calculated the B-Scores for all datasets with an assumed T-RMSE of 4.0 mmHg. Large differences between the T-RMSE and the base performances resulted in increased B-Scores. The B-Score scored highest for the “24-h Hard” dataset (as expected with constant T-RMSE; Fig. 3).

With the base-performance values calculated, we were able to calculate the B-Scores for each development dataset. Visual intuition about model performance based on base-performance values correlates with the calculated B-Scores (Fig. 3).

### B-Score under increasing standard deviation

**Figure 4:**
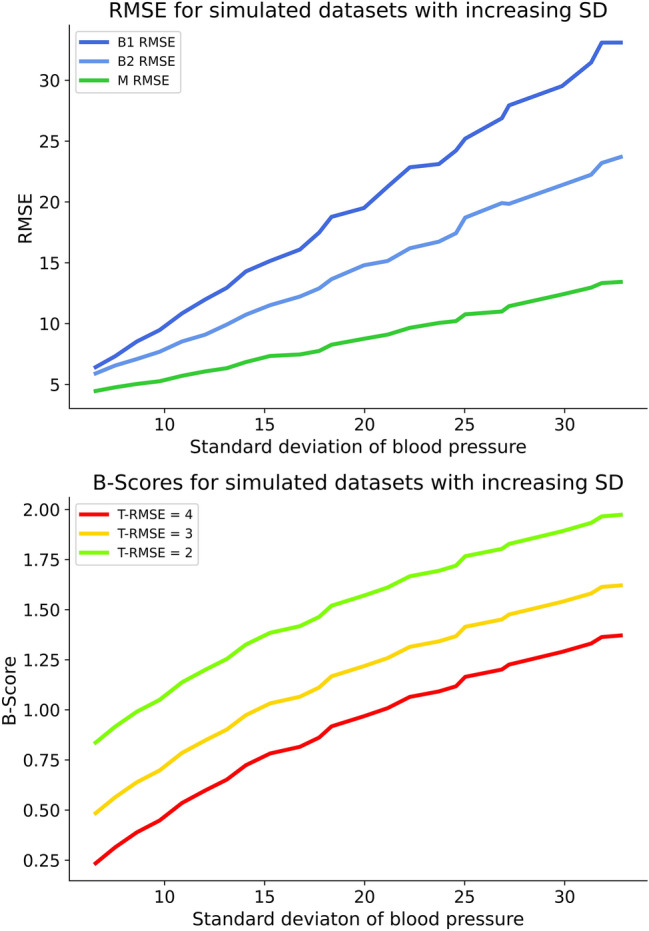
Base performances and B-Scores plotted against increasing standard deviations of simulated datasets. The upper panel shows rising base performance values (B1-, B2- and M-RMSE) for increasing standard deviation. Accordingly, the lower panel shows increasing B-Scores for constant T-RMSE values and increasing standard deviation. The B-Score discriminates between three tested T-RMSE values.

Simulating generic datasets with increasing BP standard deviation revealed a clear connection between increasing base performance values and increasing standard deviation. Subsequently, B-Scores rose with increasing BP standard deviation under constant T-RMSE values. The B-Score discriminated well between different tested T-RMSE values (Fig. 4).

### Published small dataset (dobutamine)

**Figure 5:**
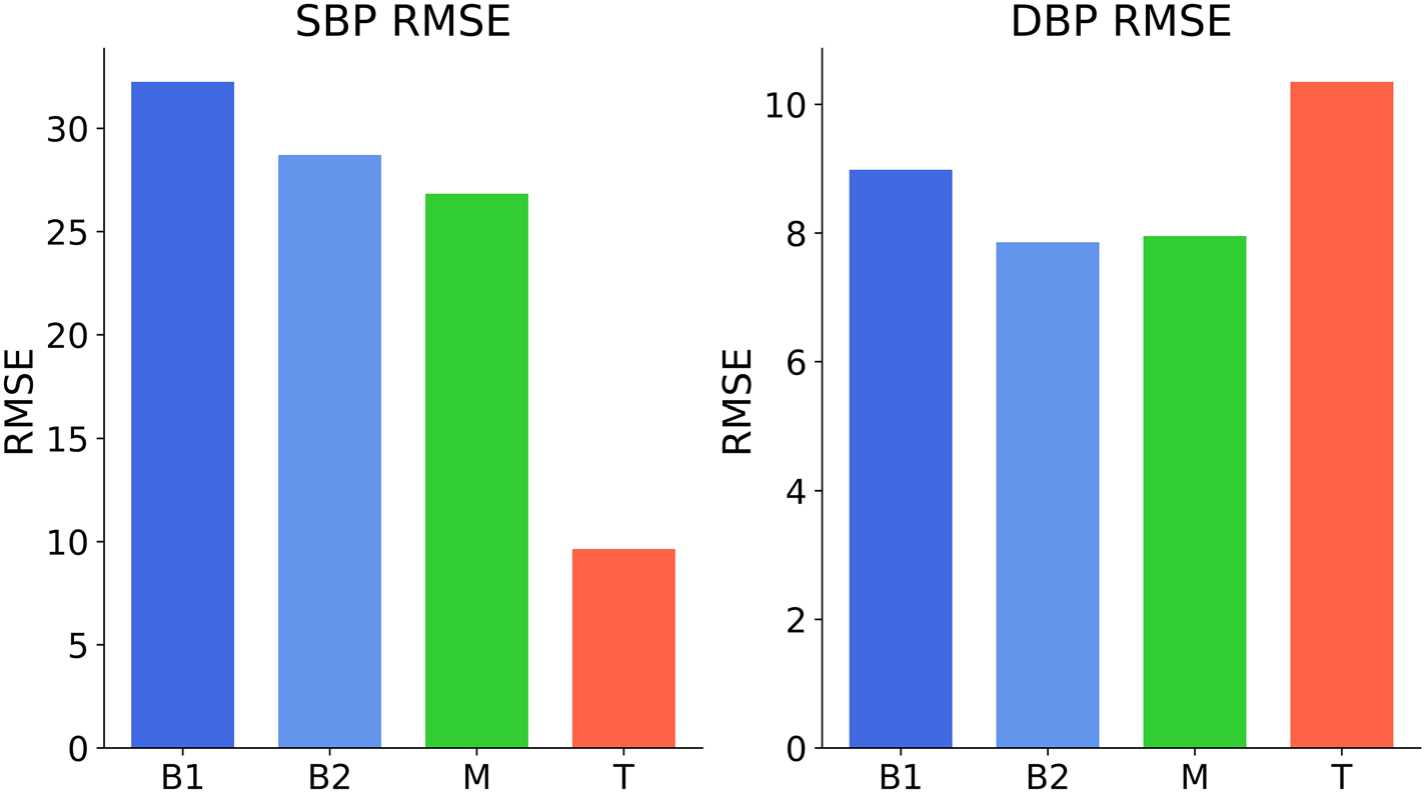
RMSE values calculated for the “dobutamine” dataset. The resulting systolic B-Score was 0.943. The diastolic B-Score is < 0.00 as the T-RMSE is not smaller than the M-RMSE. Diverging systolic and diastolic relative performances are apparent. The systolic RMSE values were B1 = 32.25, B2 = 28.71, M = 26.83, T = 9.64. The diastolic RMSE values were B1 = 8.98, B2 = 7.86, M = 7.95, T = 10.35.

We calculated the B-Score for the “dobutamine”-dataset. For diastolic values, the proposed device’s T-RMSE was larger than the M-RMSE. According to the B-Score’s definition this results in a B-Score of < 0.00. The systolic B-Score was 0.94 (Fig. 5).

### MIMIC IV dataset

**Figure 6:**
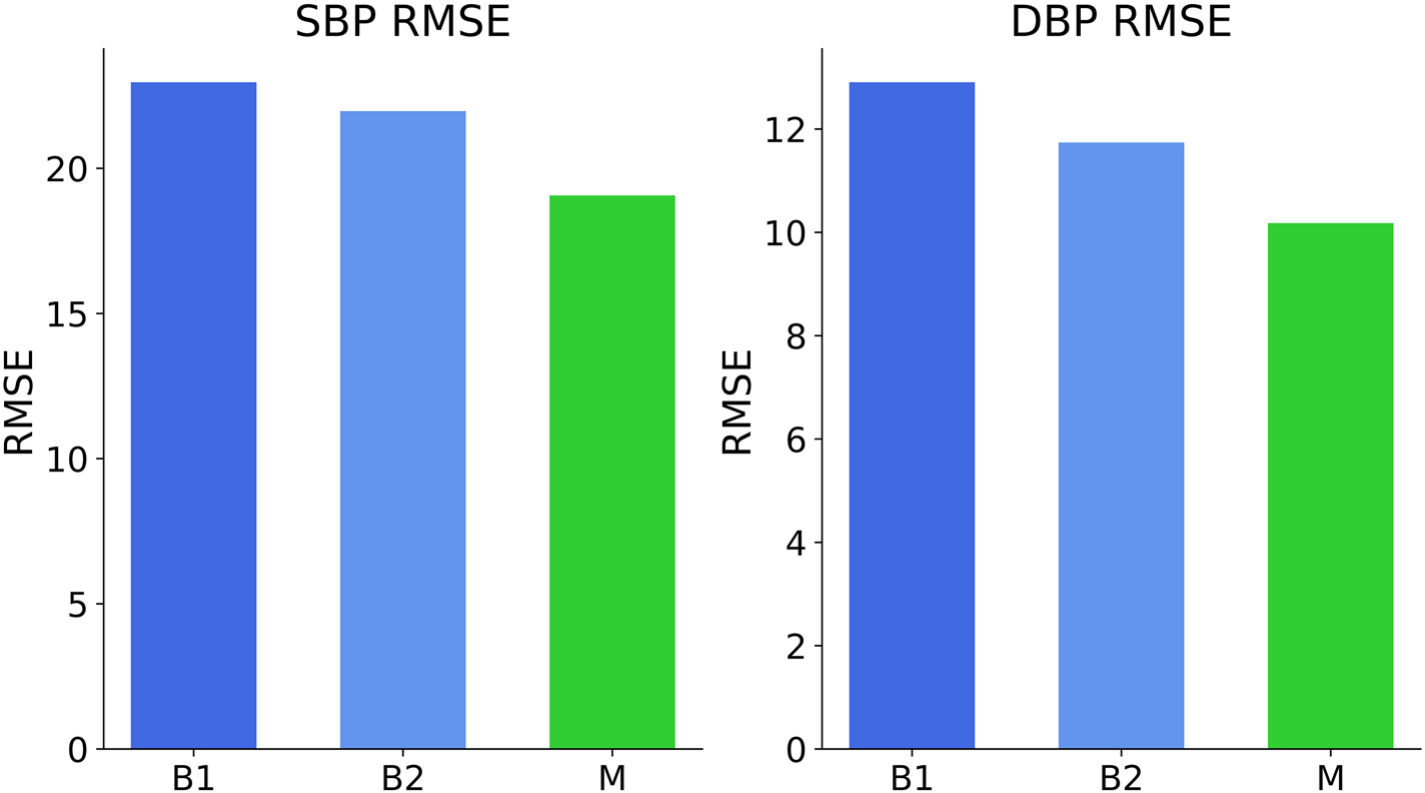
Base performances (B1-, B2-, M-RMSE) calculated for the systolic and diastolic MIMIC IV dataset. Systolic data variance is higher than diastolic. The M-RMSE shows a predictive benefit over the B1- and B2-RMSE for both systolic and diastolic values. The systolic RMSE values were B1 = 22.96, B2 = 21.97, M = 19.07. The diastolic RMSE values were B1 = 12.91, B2 = 11.74, M = 10.18.

We calculated the base performances (B1-, B2-, M-RMSE) for the systolic and diastolic MIMIC IV datasets (Fig. 6).

**Figure 7:**
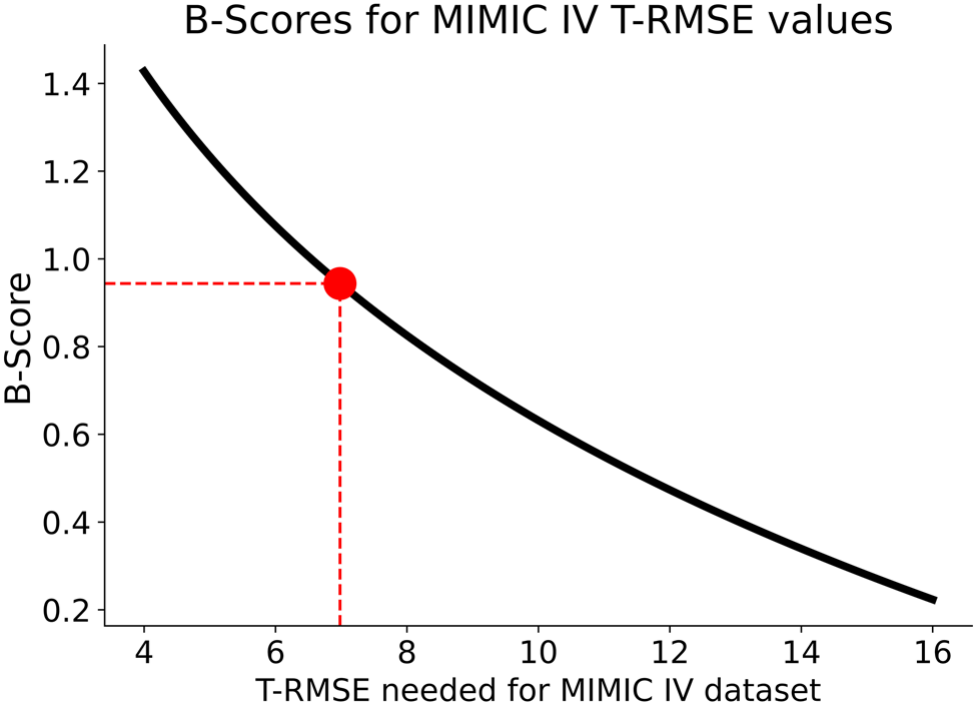
B-Scores calculated for various systolic MIMIC IV T-RMSE values (black line). The red dot indicates the T-RMSE which reaches a coequal performance to the device proposed by Patzak et al. The red dashed lines indicate the B-Score (0.96) and T-RMSE (6.98) of coequality.

We used these base performance values to calculate the systolic and diastolic T-RMSE value. A new BP estimation would need to reach a systolic T-RMSE of 6.98 on the MIMIC IV dataset to perform coequally to the system proposed in the publication of the “dobutamine” dataset^20^. Smaller T-RMSE values would indicate a novel system outperforming the proposed device (Fig. 7).

We did not calculate a diastolic T-RMSE, as any T-RMSE smaller than the MIMIC IV B1-, B2- and M-RMSE values would be sufficient. Therefore, a T-RMSE smaller than the diastolic MIMIC IV M-RMSE (10.18, Fig. 5) outperforms the device tested on the “dobutamine” dataset.

### Time complexity analysis

**Figure 8:**
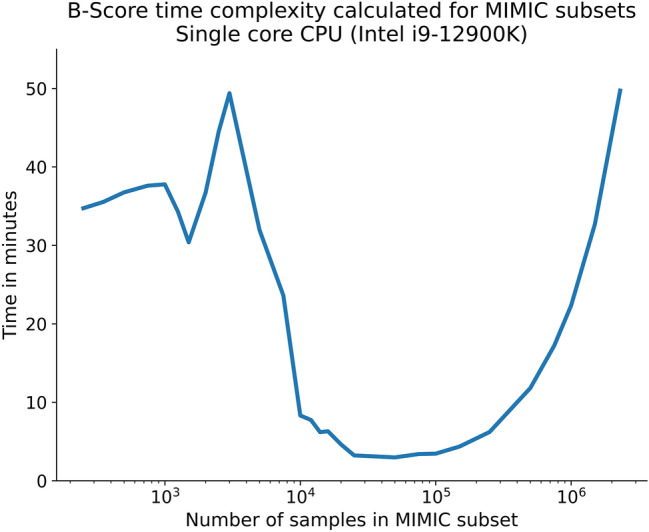
Time complexity analysis for B-Score calculation derived from MIMIC IV subsamples increasing in size. The x-axis is scaled logarithmically to allow visual interpretation.

The time complexity analysis revealed a U-shaped dependency between dataset size and time needed for B-Score calculation. Calculation times were between 3 min for medium sized datasets (50,000 BP values) and 50 min for very small (250 BP values) and extremely large (2.3 million BP samples) (Fig. 8).

On multicore processors, systolic and diastolic B-Scores can be calculated simultaneously without noticeable time delay. Noticeable, GPU acceleration does negatively affect calculation times. The standard deviation of dataset reshuffles (which are averaged for M-RMSE calculation) was below 3 mmHg for all subsamples and below 1 mmHg for all samples with more than 1250 samples.

## Discussion

The B-Score is a tool for comparing the relative performances of BP estimation systems. It sets measures of absolute model performance (regularly reported) in contrast to dataset specific parameters (base performances). It is based on the RMSE, combining insights about absolute error (cf. mean-absolute error) and measurement consistency (cf. correlation coefficient). The B-Score allows the comparison of performances between various systems tested on different datasets as higher B-Scores equal better relative performance.

We ensured reliable B-Score results and have shown its applicability by using generic datasets with differing inter- and intrapersonal BP variability. Further, the B-Score discriminated correctly between “easy” (“Lab”) and “hard” (“24 h Hard”) datasets, for a set fictional T-RMSE (= same absolute performance for all datasets). Further, the B-Score showed expected results for generic datasets with increasing BP standard deviation. It discriminated between different tested T-RMSE values for increasing base performance values.

To test the B-Score in real-world data, we used a small, published dataset with a proposed device for BP estimation (“dobutamine” dataset) and the to our knowledge largest available BP dataset (“MIMIC IV”). We calculated the B-Score for the “dobutamine” dataset and retrieved greatly differing results for systolic and diastolic performance. The B-Score revealed a markedly better systolic performance even though the absolute performance measures were largely the same between systolic and diastolic values. This illustrated the important additional information provided by a measure of relative performance (B-Score).

Further, we calculated the base performances (B1-, B2-, M-RMSE) for the MIMIC IV dataset. These parameters allowed us to calculate the T-RMSE value a new system would need to reach on the MIMIC IV dataset to provide coequal performance to the device tested by Patzak et al. (“dobutamine” dataset). The inter- and intrapersonal BP variability in the MIMIC IV dataset was smaller than in the “dobutamine” dataset. Consequently, the needed T-RMSE to reach coequal relative performance was lower than the one derived from the “dobutamine” dataset.

This analysis revealed important insights into the described datasets but more importantly proved that the B-Score is easily calculatable even in extreme (comparing very small vs. very large datasets) real-world use cases. This is further underlined by the quick calculation times which allows to derive the B-Score within one hour on using a modern CPU. This is true for virtually all dataset sizes, with minimum calculation times for medium-sized datasets. The U-shaped time complexity curve is product of increasing calculation time per training episode for larger datasets and simultaneous reduction of repeated and reshuffled calculations due to increased trust in result reliability. We consider the resulting calculation times reasonable, especially noting that the calculation time needed only applies once per dataset, as the base performances can be used to recalculate the B-Score for any new model using the same dataset within seconds.

Additionally, time complexity analysis revealed narrow standard deviations between reshuffles, even for small datasets. This supports the assumption the B-Score calculation generates reliable and repeatable results.

We designed the B-Score to be intuitively understandable (higher B-Scores equal better relative performance) and easily calculable. Any researcher using a modern machine can calculate the B-Score for their data within 1 workday using the provided code (Supplementary Appendix 2).

As the B-Score is partly determined by inter- and intrapersonal BP variability within a given dataset, researchers aiming for high B-Scores are incentivized to develop their models for high variability datasets. This is important, because systems which perform well on heterogenous data are more likely to generalize to real-world applicability.

We envision investigators in the field of BP estimation devices to calculate base performance (B1-, B2-, M-RMSE) and B-Score values for their respective datasets and proposed systems. This will allow intuitive comparability between the plethora of systems available and under development.

We did not create the B-Score to replace validation studies and standardized validation protocols. These serve an important role in guaranteeing methodological comparability, which cannot be displaced by the B-Score.

We anticipate the B-Score to be an important tool for systems and devices which have not yet reached the stage of full-on clinical validation. It empowers researchers and engineers to quickly assess their system’s relative performance on whichever dataset they have available. Researchers will be able to detect promising trends in the scientific literature more quickly and securely when B-Score are reported in the scientific literature during early stages of development. Further, the B-Score can become a tool for advanced, post-validation system testing. It allows to compare performances for distinct groups (e.g., pregnant women, children, etc.) or under special circumstances (e.g., sport) which are not covered by validation protocols.

## Conclusion

The B-Score is a novel, functional measure of relative BP estimation performance. We proved its reliable results, ease of calculation even for large datasets and desired properties with generated datasets. Followingly, the B-Score revealed important insights in an extreme, real-world use case (comparing a very small vs. very large dataset).

The B-Score is easily interpreted and quickly calculated for any given dataset. We envision the B-Score to be used in pre-validation studies for system development and in advanced, post-validation analyses for special subgroups or measurement circumstances.

We hope that the B-Score will in the future become a useful and broadly applied tool for model performance comparison. It enables the quick and secure detection of promising trends in the scientific literature and allows scientists and engineers to quickly assess the performance of their systems.

## Supplementary Information

Supplementary Information 1.

Supplementary Information 2.

## Data Availability

The “dobutamine” dataset is a re-analysis of already published data (Ref.^20^) and is available from the corresponding author of this publication upon reasonable request. The MIMIC IV dataset is available following the instructions from reference 25. The Code mentioned in the article is openly available in a public repository (Supplementary Appendix 2). For further information about data availability, please contact the corresponding author.
