# Supplementary material for: The B-Score is a novel metric for measuring the true performance of blood pressure estimation models
Source: Sci Rep. 2022 Jul 16;12:12173. doi: 10.1038/s41598-022-16527-2 (PMC9288457; doi:10.1038/s41598-022-16527-2)
Supplement: Supplementary file 1 — Supplementary Information 1. [file 41598_2022_16527_MOESM1_ESM.docx]

Appendix 1

| **Base performance (M) DL architecture parameters** | |
| --- | --- |
| *Layers* |  |
| *Dense (fully connected)* |  |
| Units | 8, 6, 4, 3, 1 |
| Activation | Rectified Linear Unit (ReLU) |
| *Batch Normalization* | after each Dense layer |
|  |  |
| *Compiler* |  |
| *Optimizer* | Adam |
| Learning rate | 0.001 |
| Beta 1 | 0.9 |
| Beta 2 | 0.999 |
| Epsilon | 1e-07 |
| Amsgrad | False |
| *Loss* | Mean squared error |
|  |  |
| *Fit* |  |
| Batch size | 256 |
| Epochs | > 10,000 |
|  |  |
| *Callbacks* |  |
| *Early Stopping* |  |
| Monitor | Validation loss |
| Patience | Min (200, Max (2, (200,000 / dataset rows))) |
| Reduce Learning Rate on Plateau |  |
| Monitor | Loss |
| Factor | 0.1 |
| Patience | Replaces Early Stopping for 5 iterations (see provided Code) |
| *Model Checkpoint* |  |
| Monitor | Validation loss |
| Save frequency | Every epoch |
| Save best only | True |

Appendix 1: Hyperparameters for basic Deep Learning model (M) construction. Parameters must not be changed to preserve outcome comparability and generalization. For precise information about k-fold validation and sampling procedures please refer to the code in App. 3.

Appendix 2

The B-Score is a novel metric for measuring the true performance of blood pressure estimation models
Supplementary Code for the B-Score manuscript submitted to Scientific Reports. The supplied code helps researchers to quickly calculate the B-Scores for their respective blood pressure estimation algorithms.

Github: <https://github.com/ChariteBothe/Scientific_Reports_B-Score-Code>

Appendix 3

$$\Delta_{short} ={amplitude}_{short} \cdot\sin\left( t \cdot\frac{2\pi}{10s} \right)$$

$$\Delta_{long} = {amplitude}_{long} \cdot\left( -\cos\left( (t- t_{circ min}) \cdot\frac{2\pi}{86400s} \right) \right)$$

$$BP= \Delta_{short}+\Delta_{long}+{BP}_{mean, pat.}+measurement uncertainty$$

*Where* $\Delta_{short}$ *= short term fluctuation,* $t$ *= timepoint of measurement in seconds,* $\Delta_{long}$ *= long term fluctuation,* $t_{circ min}$ *= timepoint of minimal BP in seconds,* ${BP}_{mean, pat.}$ *= Mean BP for given subject,* $measurement uncertainty$ *= device specific measurement uncertainty*

|  | **SBP** | | | **DBP** | | |
| --- | --- | --- | --- | --- | --- | --- |
|  | *Normal* | *Hard* | *Lab* | *Normal* | *Hard* | *Lab* |
| *amp. _short_* | 2.5 (1.0) | 2.5 (1.0) | 2.5 (1.0) | 2.5 (1.0) | 2.5 (1.0) | 2.5 (1.0) |
| *amp. _long_* | 7.5 (2.0) | 12.0 (4.0) | 7.5 (2.0) | 5.0 (2.0) | 8.0 (4.0) | 5.0 (2.0) |
| ${BP}_{mean, pat.}$ | 120.0 (10.0) | 120.0 (20.0) | 120.0 (6.0) | 80.0 (8.0) | 80.0 (12.0) | 80.0 (5.0) |
| *meas. uncert.* | 0.0 (3.0) | 0.0 (7.0) | 0.0 (3.0) | 0.0 (3.0) | 0.0 (7.0) | 0.0 (3.0) |
| $t_{circ min}$ | 3.0 (1.0) | 3.0 (1.0) | 3.0 (1.0) | 3.0 (1.0) | 3.0 (1.0) | 3.0 (1.0) |

Table: Parameters used for generic dataset creation. Parameters are defined as normally distributed values with their mean and SD (in brackets) values provided in the table. Parameters are re-sampled for every generated patient, resulting in patient specific time (t)-dependent BP functions. *amp. = amplitude, meas. Uncert. = measurement uncertainty,* ${BP}_{mean, pat.}$ *= Mean BP for given subject,* $t_{circ min}$ *= timepoint of minimal BP (here in hours)*

Appendix 4

$$T RMSE= \sqrt[4]{\frac{B1 RMSE\cdot B2 RMSE \cdot{M RMSE}^{2}}{{10}^{(2 \cdot B Score)}}}$$

Appendix 5


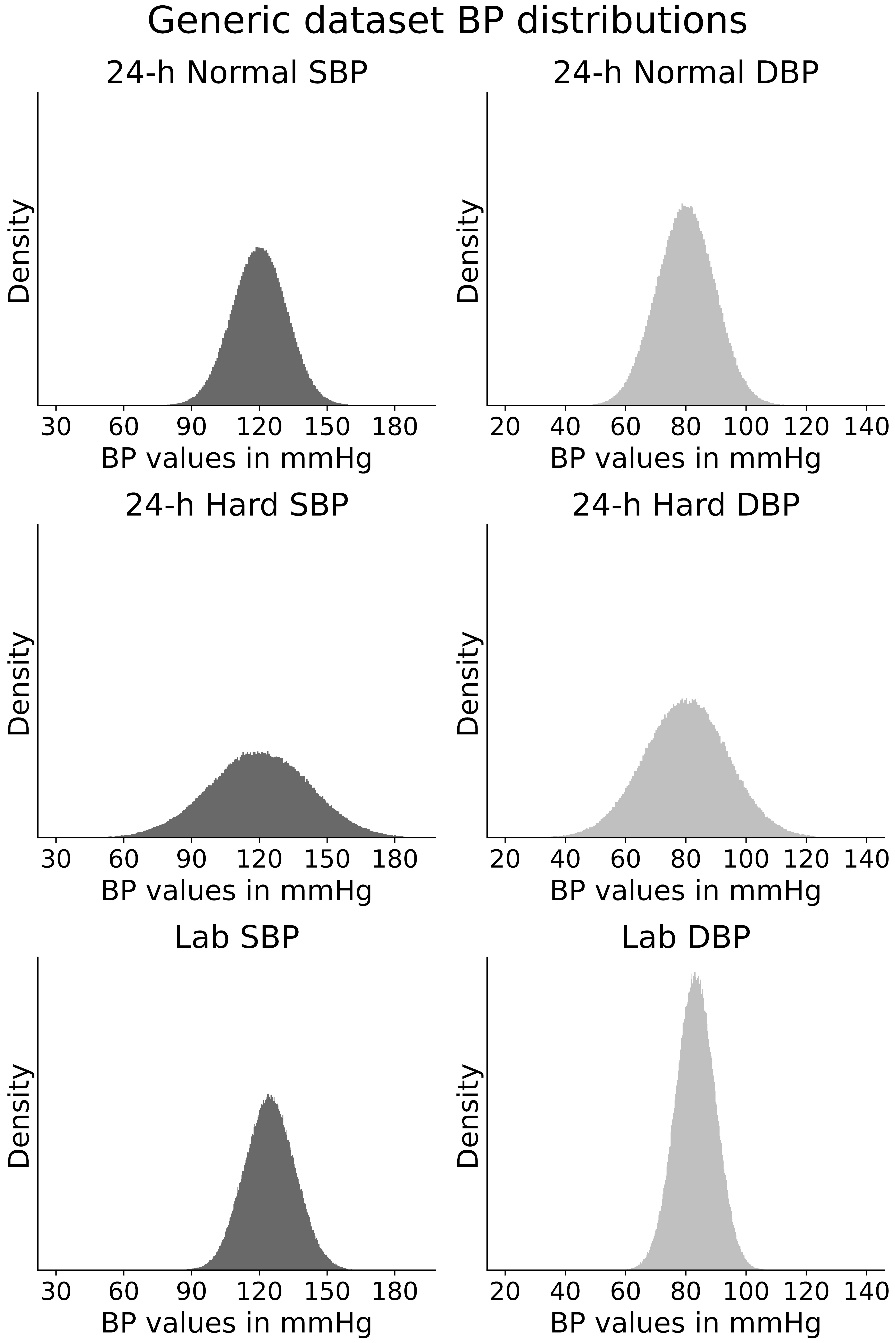


Appendix 5: BP density distributions for the six generic datasets. Each dataset consists of 500,000 samples.
